# Supplementary material for: Tracing back primed resistance in cancer via sister cells
Source: Nat Commun. 2024 Feb 7;15:1158. doi: 10.1038/s41467-024-45478-7 (PMC10850087; doi:10.1038/s41467-024-45478-7)
Supplement: Supplementary file 1 — Supplementary Information [file 41467_2024_45478_MOESM1_ESM.pdf]

Supplementary Information

Tracing back primed resistance in cancer via sister cells

The PDF file includes:

Supplementary Figures

Supplementary Figure 1-10

Supplementary Note

Computer simulation of the ReSisTrace experiment

Supplementary Methods

Flow cytometry analysis

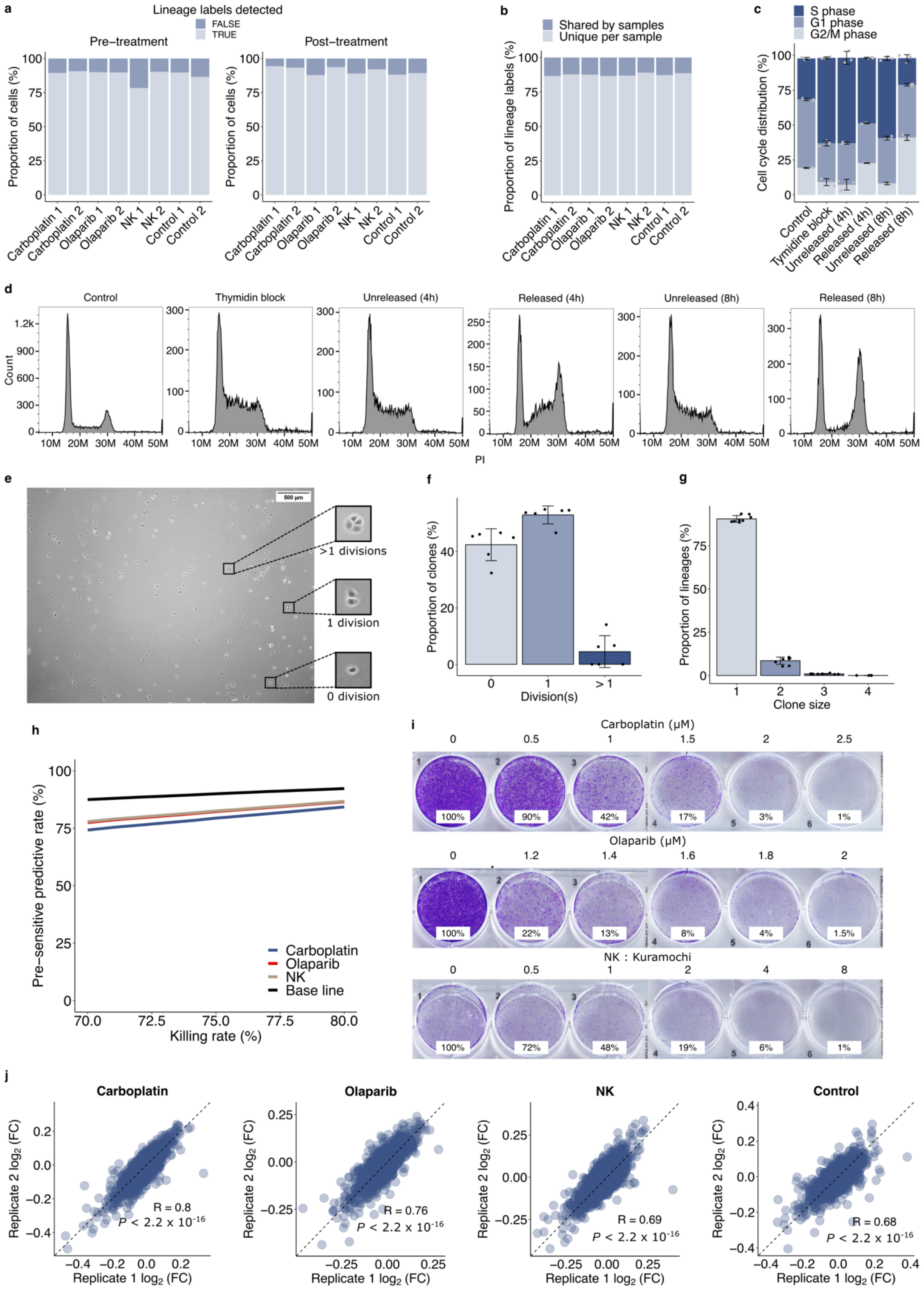
a
Lineage labels detected
FALSE
TRUE
Pre−treatment
Post−treatment
Proportion of cells (%)
Carboplatin 1
Carboplatin 2
Olaparib 1
Olaparib 2
NK 1
NK 2
Control 1
Control 2
b
Shared by samples
Unique per sample
Proportion of lineage labels (%)
c
S phase
G1 phase
G2/M phase
Cell cycle distribution (%)
Control
Tymidine block
Unreleased (4h)
Released (4h)
Unreleased (8h)
Released (8h)
d
Control
Thymidin block
Unreleased (4h)
Released (4h)
Unreleased (8h)
Released (8h)
Count
PI
e
500 μm
>1 divisions
1 division
0 division
f
Proportion of clones (%)
Division(s)
g
Proportion of lineages (%)
Clone size
h
Pre−sensitive predictive rate (%)
Killing rate (%)
Carboplatin
Olaparib
NK
Base line
i
Carboplatin (μM)
100%
90%
42%
17%
3%
1%
Olaparib (μM)
100%
22%
13%
8%
4%
1.5%
NK : Kuramochi
100%
72%
48%
19%
6%
1%
j
Carboplatin
Olaparib
NK
Control
Replicate 2 log2 (FC)
Replicate 1 log2 (FC)
R = 0.8
P < 2.2 x 10-16
R = 0.76
P < 2.2 x 10-16
R = 0.69
P < 2.2 x 10-16
R = 0.68
P < 2.2 x 10-16

Supplementary Fig. 1 Cell doubling, lineage labelling efficiency, and quality assessment of ReSisTrace. (**a**) Percentage of cells with the lineage barcodes detected in each sample; numbers 1 and 2 refer to repeats within each condition. (**b**) Proportion of sample-shared or sample-unique barcodes in each pre-treatment sample. (**c**) Cell cycle phase proportions after 42 hours thymidine block, and 4 or 8 hours following release or control without release, and (**d**) respective histograms from flow cytometry. (**e**) A representative phase-contrast image of cells after one doubling time of 48 hours, and (**f**) proportion of clones for indicated numbers of divisions. Error bars are mean +/- s.d. of six images. (**g**) Proportion of lineages with indicated clone sizes detected in the pre-treatment scRNA-seq samples. Error bars are mean +/- s.d. of eight samples (4 treatment conditions each with 2 replicates). (**h**) Pre-sensitive predictive rate for each treatment condition, as compared to the upper bound of perfect doubling and no drop-out (baseline). (**i**) Colony formation of Kuramochi cell line treated by NK cells (1 day), olaparib (7 days), or carboplatin (3 days) with indicated ratio or concentration, followed by 7, 6, or 10 days recovery, respectively. The number under each well indicates the remaining cell number compared to the corresponding untreated control well. (**j**) Consistency of gene expression changes for pre-resistant against pre-sensitive cells between the replicates of each treatment condition: carboplatin (1.2 μM, 3 days), olaparib (1.2 μM, 7 days), and NK cells (26,000 NK cells to 16,000 cancer cells, 1 day). (Carboplatin n = 30,613 genes; Olaparib n = 30,586 genes; NK n = 30,613 genes; Control n = 27,478 genes). The Pearson's correlation and its two-tailed test P value are shown. Source data are provided as a Source Data file.

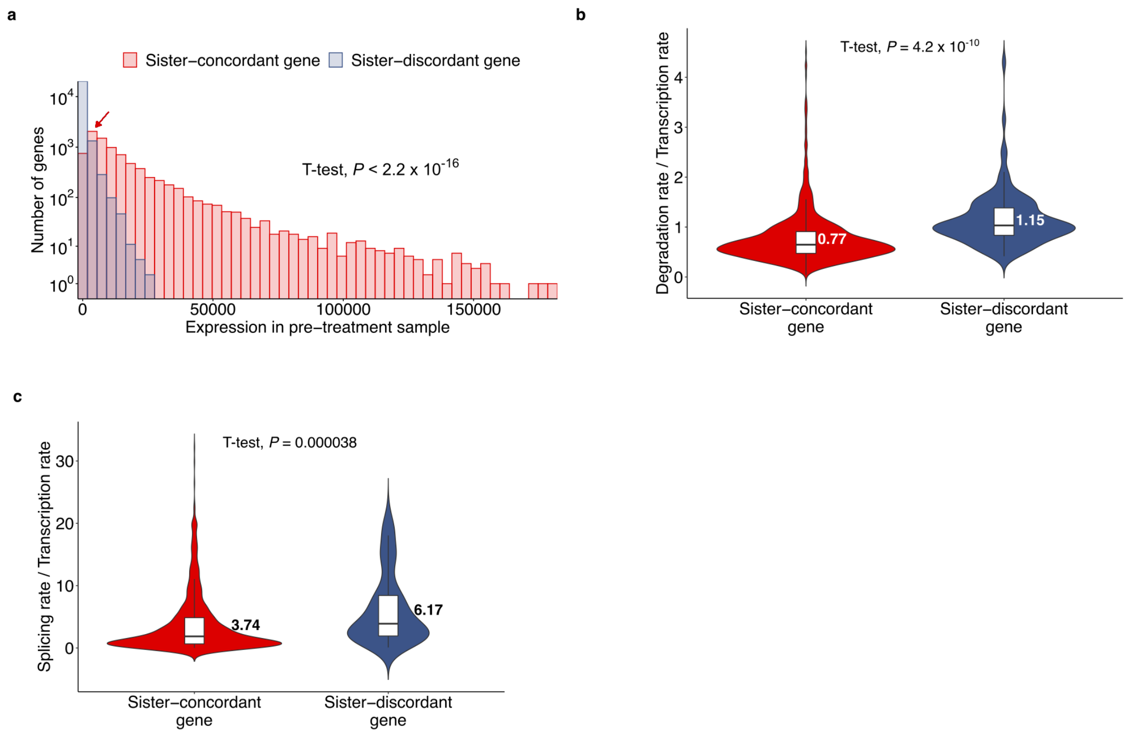

Supplementary Fig. 2 Characteristics of sister-concordant and sister-discordant genes. (**a**) Histogram showing the number of genes at different expression levels of sister-concordant (red, n = 8,419) or sister-discordant (blue, n = 21,896) genes in the pre-treatment samples. Red arrow indicates the bin ([1839.89, 5519.11]) used to determine the subsets of sister-concordant and sister-discordant genes in the RNA velocity and pathway enrichment analyses. (**b**, **c**) Average RNA degradation to transcription ratio and average RNA splicing to transcription ratio of sister-concordant (n = 919) and discordant genes (n = 107) velocity genes used to fit the dynamical model. Two-tailed t-test P values are shown. The boxplots show the 25th percentile, median, and 75th percentile, with the whiskers indicating the 1.5× interquartile range. Source data are provided as a Source Data file.

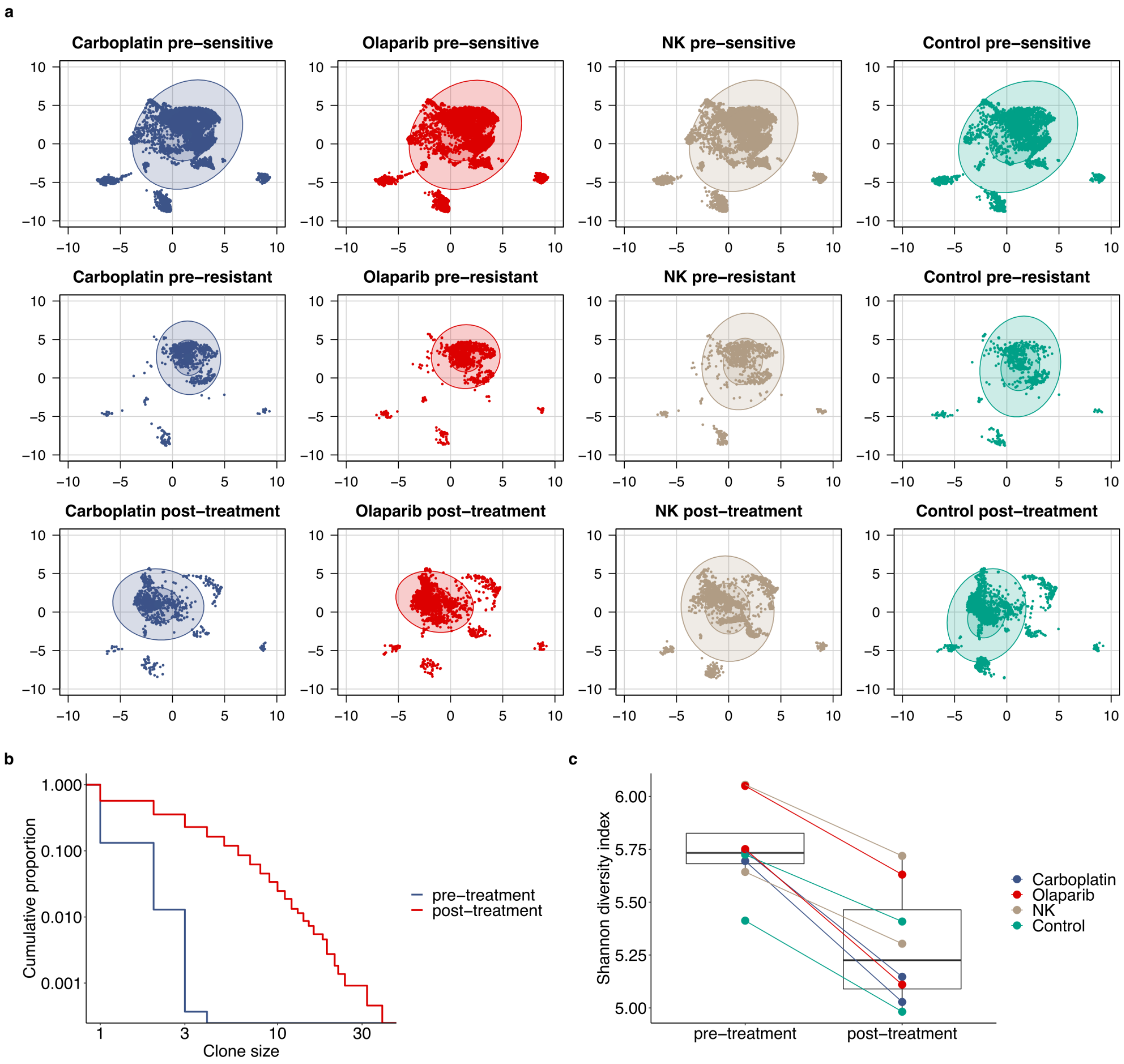

Supplementary Fig. 3 Diversity of ReSisTrace populations. (**a**) UMAP projection of pre-sensitive, pre-resistant, and post-treatment cells for each treatment. (**b**) Cumulative proportion of lineages with different clone sizes in the pre-treatment or post-treatment samples. (**c**) Shannon diversity index in the pre-treatment or post-treatment samples for each replicate in different treatment conditions. The boxplots show the 25th percentile, median, and 75th percentile, with the whiskers indicating the 1.5× interquartile range. Source data are provided as a Source Data file.

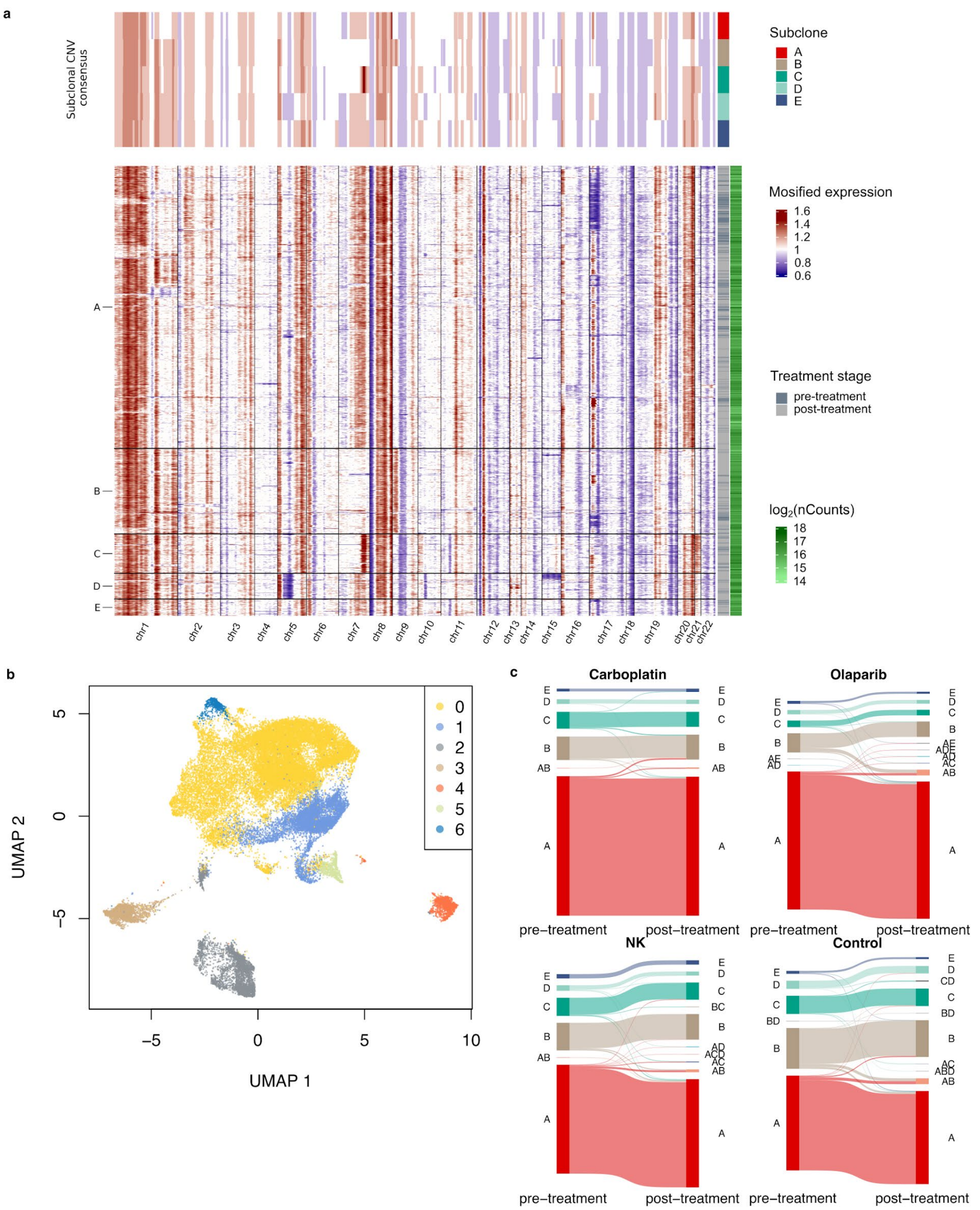

Supplementary Fig. 4 Lineages maintain their subclonal identities during the treatment. (**a**) Heatmap showing the subclonal CNV profiles of the carboplatin samples inferred from their scRNA-seq data. The lower panel shows the CNV profiles for the individual cells. The upper panel shows the consensus CNV profiles for each identified subclone. (**b**) Unsupervised Leiden clustering of transcriptomic profiles projected on the UMAP; adjusted Rand index between transcriptomic clusters and inferred subclones is 0.83. (**c**) Sankey plot for the subclonal dynamics during each treatment. Each subclone is represented by a colored band, the height of which corresponds to the proportion of identified lineages. Source data are provided as a Source Data file.

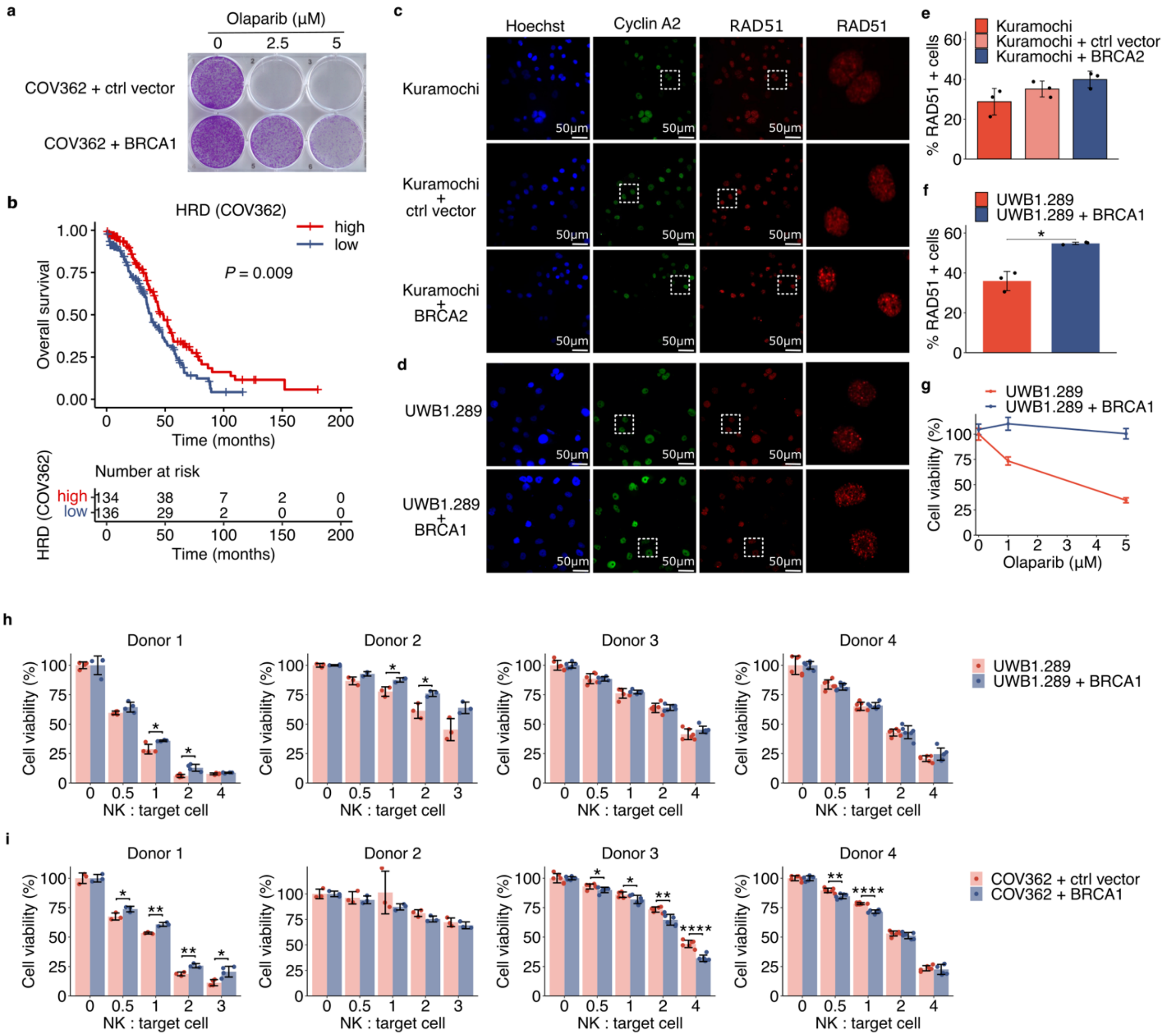

Supplementary Fig. 5 BRCA restored cell lines display an HRP phenotype and are less sensitive to NK killing. (**a**) Colony formation assay of parental and BRCA1 restored COV362 cells treated with indicated concentrations of olaparib. (**b**) Kaplan-Mayer curves for the overall survival in the TCGA ovarian cancer cohort, stratified to high and low groups by the experimental HRD signature (COV362) (hazard ratio = 1.51, *P* = 0.009, log-rank test). The genes in the signatures were selected by adjusted *P* value threshold of 1×10^{-8}. (**c-f**) HRD test showing RAD51 foci for control plasmid transduced or BRCA2 restored Kuramochi cells (**c**, **e**), and UWB1.289 parental or BRCA1 restored cells (**d**, **f**) Error bars are mean +/- s.d. of three replicates. * *P* = 0.0198, two-tailed t-test (**g**) Olaparib sensitivity of UWB1.289 parental or BRCA1 restored cells. * 0.1 < *P* < 0.05, two-tailed t-test. Error bars are mean +/- s.d. of three replicates. (**h-i**) NK killing of parental or BRCA1 restored UWB1.289 cells (**h**), and control plasmid transduced or BRCA1 restored COV362 cells (**i**). Killing rates of NK cells from four different donors are shown separately. * 0.01 < *P* < 0.05; ** 0.001 < *P* < 0.01; *** 1×10^{-4} < *P* < 0.001; **** *P* < 1×10^{-4}, two-tailed t-test (exact *P* values are listed in Source Data file). Error bars are mean +/- s.d. of at least three replicates. Source data are provided as a Source Data file.

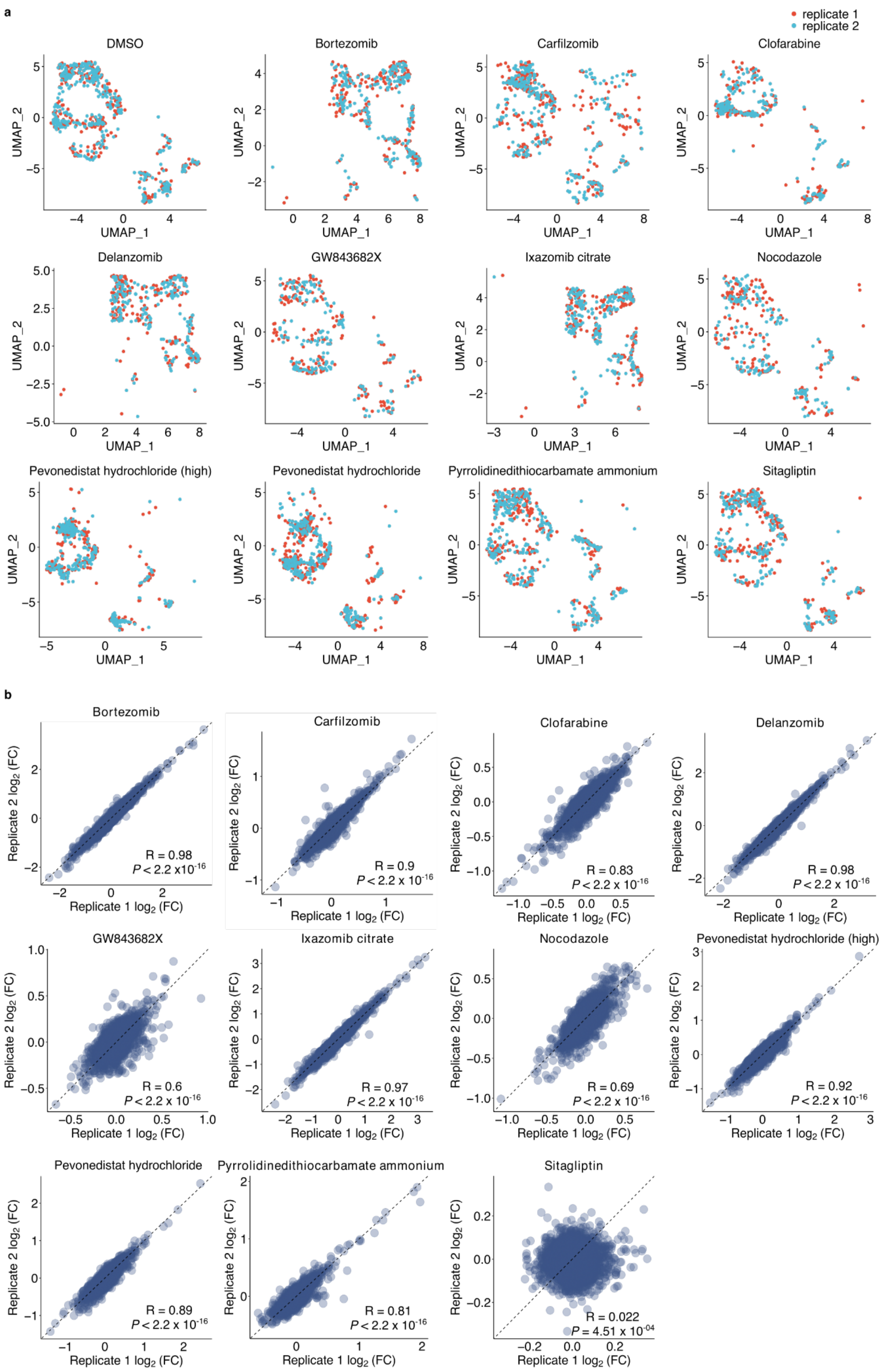

Supplementary Fig. 6 Drug-induced expression profiles shown as UMAP projection (**a**), and gene expression changes to the DMSO control for corresponding replicates 1 and 2 of each drug treatment (**b**). The Pearson's correlation and its two-tailed test *P* value are shown. Source data are provided as a Source Data file.

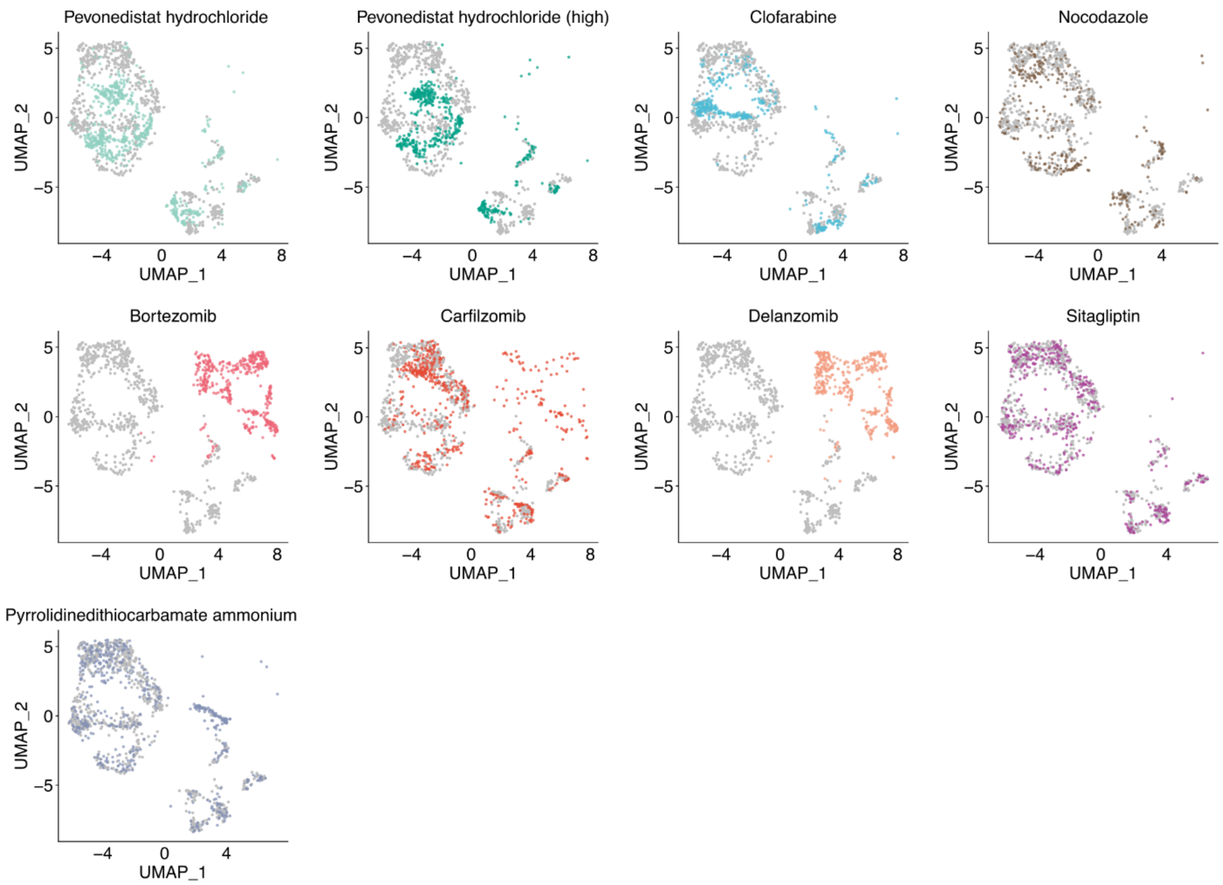

Supplementary Fig. 7 Single-cell gene expression of drug perturbed Kuramochi cells as UMAP projections, with indicated drug-induced cells as coloured and DMSO control as grey.

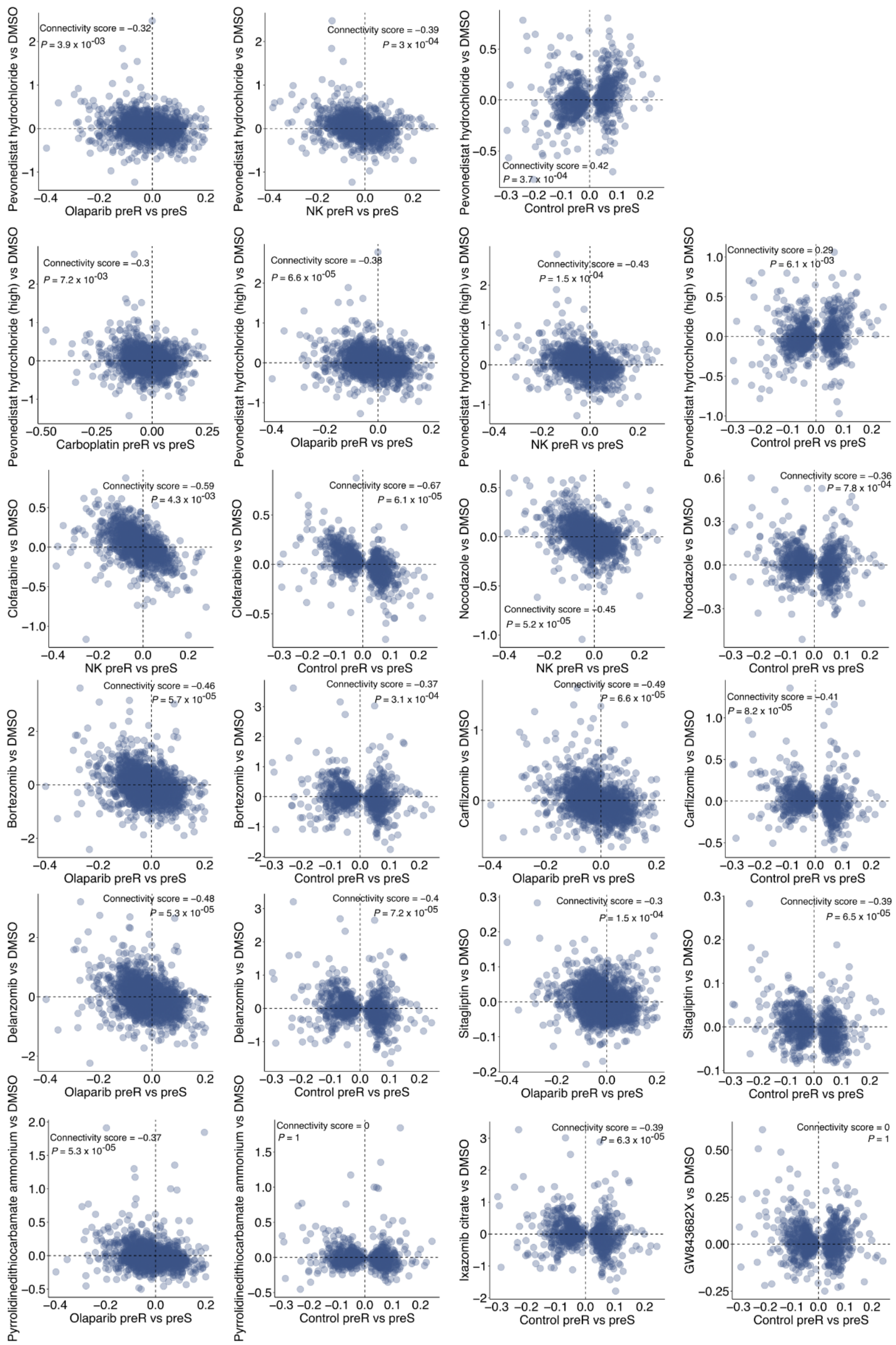

Supplementary Fig. 8 Expression changes in Kuramochi cells treated with predicted pre-sensitising drugs, compared to respective pre-resistance profiles. Y axis shows mean drug induced log_2FC against DMSO

from scRNA-sequenced Kuramochi cells, X axis shows mean PreR to PreS log_2FC in respective condition. *P* values were determined by a two-tailed permutation test. Source data are provided as a Source Data file.

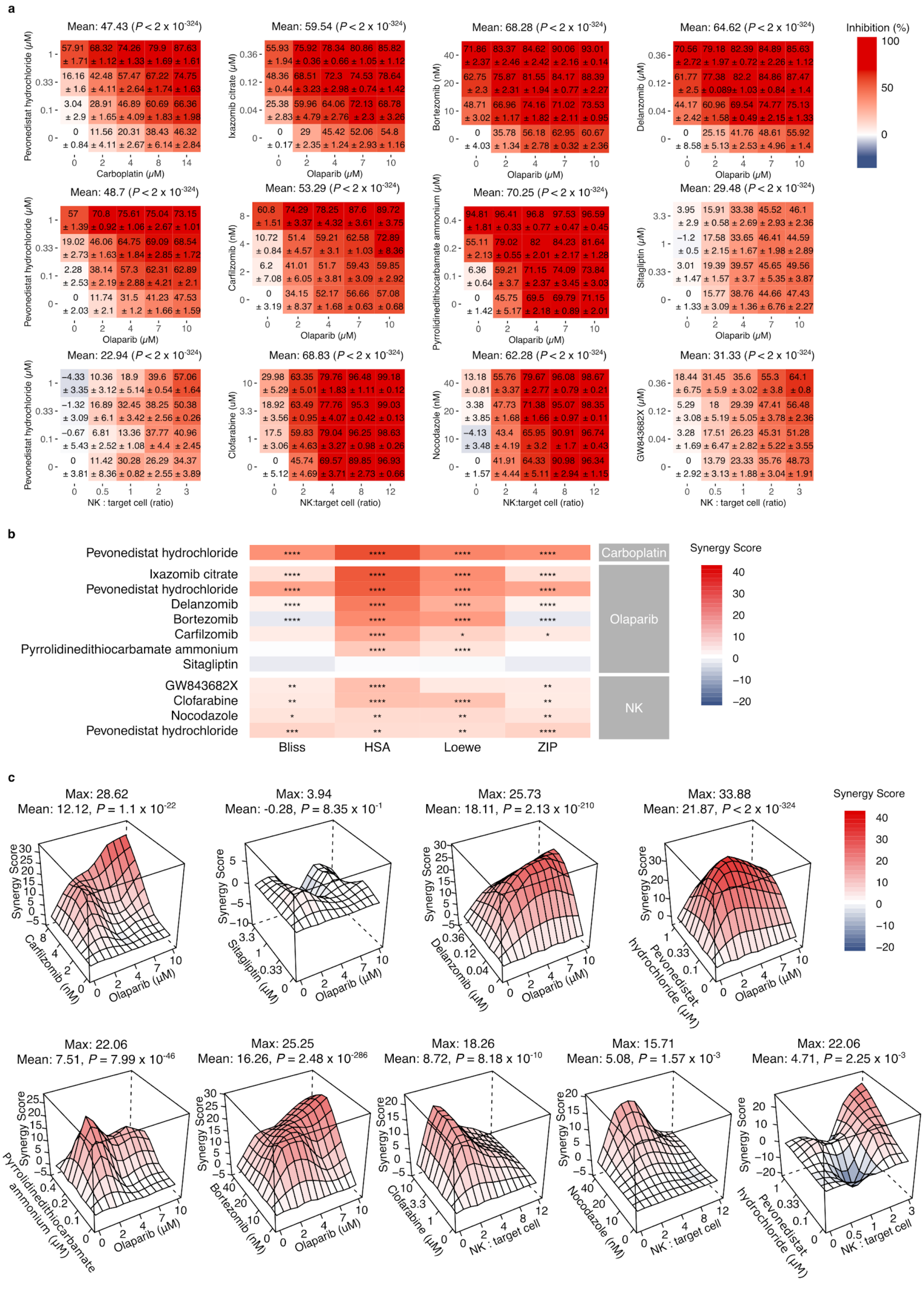

Supplementary Fig. 9 Screening results for the predicted sequential drug combinations. (a) Dose-

response matrices for the predicted sequential combinations, where cells were first treated with drugs with concentrations indicated on the vertical axis for 24 hours, followed by the treatment indicated on the horizontal axis (carboplatin or olaparib for 5 days, or by NK killing for 24 hours). *P* values are determined by two-tailed bootstrapping tests. (**b**) Synergy scores for the predicted drug combinations. * $0.01 < P < 0.05$; ** $0.001 < P < 0.01$; *** $1\times10^{-4} < P < 0.001$; **** $P < 1\times10^{-4}$, two-tailed bootstrapping test. (**c**) HSA synergy landscapes for other predicted drug combinations. *P* values are determined by two-tailed bootstrapping tests. Source data are provided as a Source Data file.

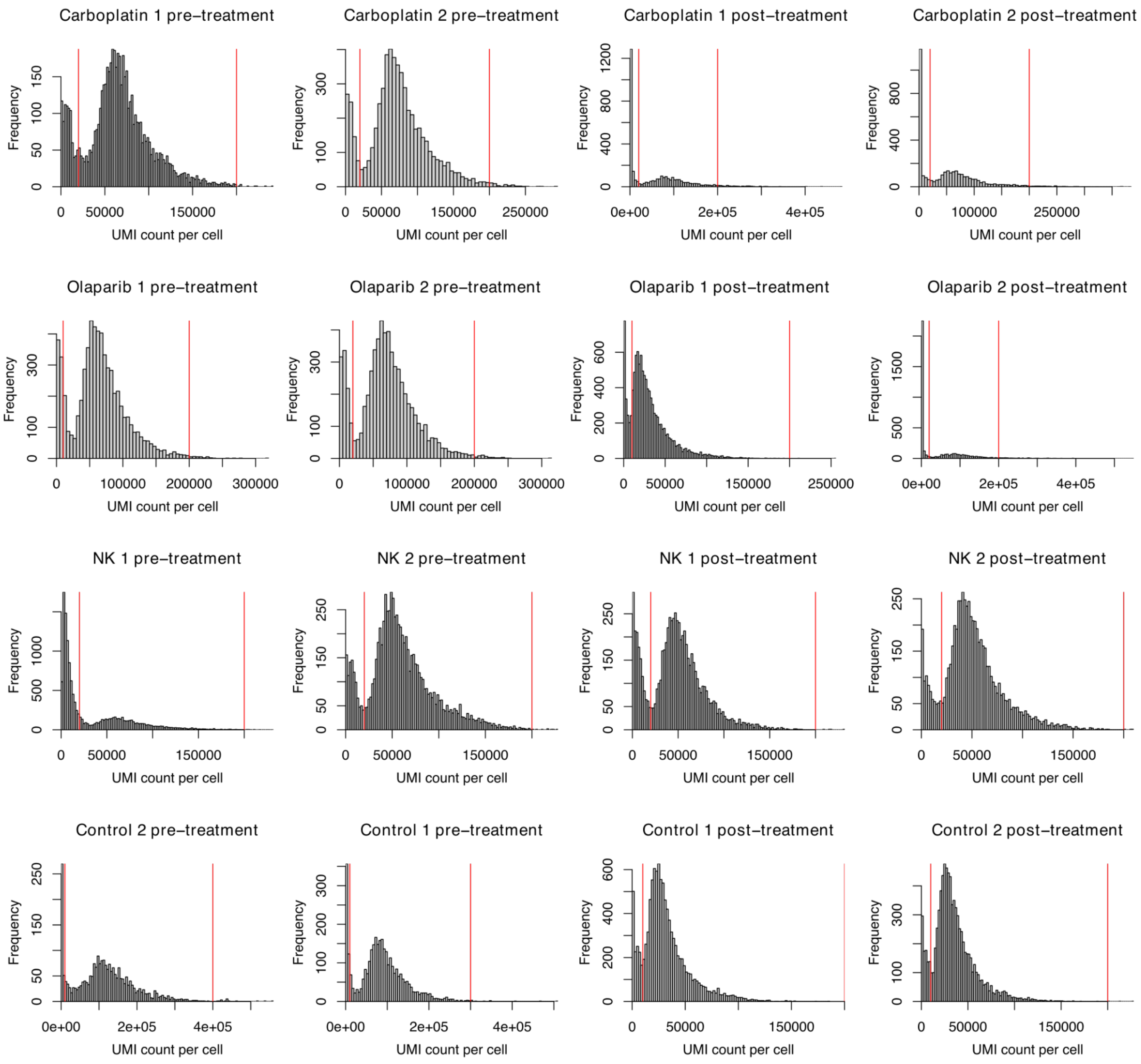

Supplementary Fig. 10 Histogram of UMI counts, showing the selected thresholds, listed in Supplementary Data 10, as red vertical lines.

Supplementary Note

Computer simulation of the ReSisTrace experiment

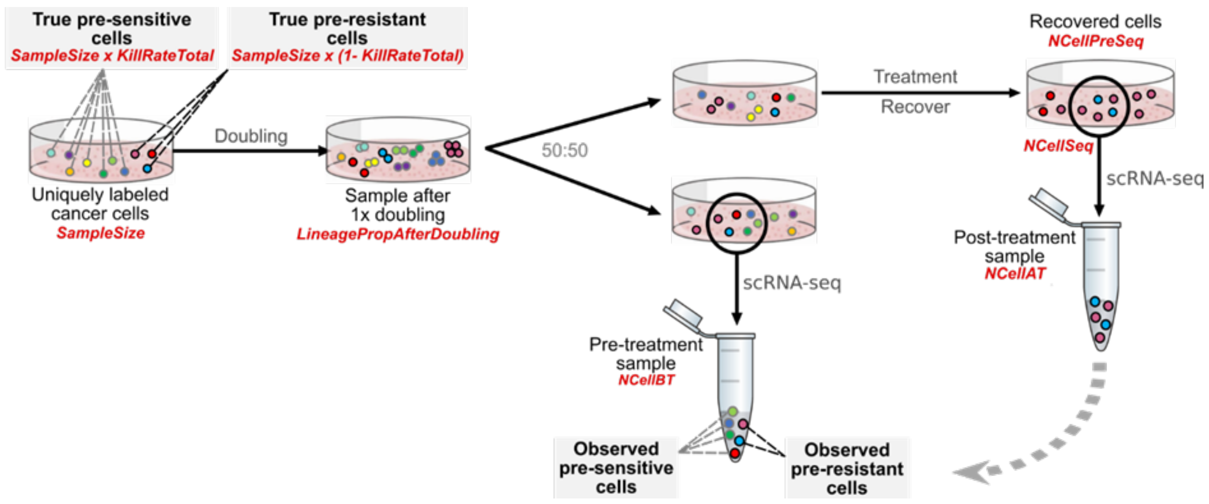

Supplementary Fig. 11 Schematic of the computer simulation of the ReSisTrace experiment.

We established a computer simulation that follows the procedure of the performed ReSisTrace experiment to assess the accuracy of observed "pre-resistant" and "pre-sensitive" categories in the pre-treatment sample (Supplementary Fig. 11). To incorporate uncertainties in the experiment, we considered a set of parameters. Initially, we simulated a pool of uniquely labelled cancer cells that started with a fixed sample size. In the simulation we set the parameter SampleSize to be 16,000, based on the number of cells used in the actual experiment. In the actual experiment, we controlled the killing rate of the anti-cancer treatment to be between 70% and 80%, which was used to set the value of KillRateTotal parameter in the simulation. From the uniquely labeled cells, we randomly selected $(\mathrm{SampleSize} \times \mathrm{KillRateTotal})$ cells as "true pre-resistant" cells, while the other $\mathrm{SampleSize} \times (1 - \mathrm{KillRateTotal})]$ cells were labelled as "true pre-sensitive" cells.

After the initialization, the cells went through the doubling stage in the simulation. Due to the experimental uncertainty, not all the cells were doubled. According to the image shown in Fig. S1c, we observed that 53.04% of the cells were doubled, 4.5% of the cells were quadrupled, and the remaining 42.46% of the cells remained as singletons in the real experiment. These proportions were modelled as the LineagePropAfterDoubling parameter in the computer simulation, which determined randomly whether a lineage had one, two or four copies.

After the doubling stage, the sample was randomly split into two samples of equal sizes in the simulation. One sample was profiled with single-cell RNA sequencing (scRNA-seq), while the other one was profiled after anti-cancer treatment and recovery. For the sample that directly went for scRNA-seq, not all the cells were successfully sequenced due to the quality control step. We defined the number of cells that pass the quality control as the parameter NCellBT, ranging between 4,748 and 5,851 for the three treatment conditions, and was estimated from the actual results of the scRNA-seq data shown in Supplementary Data 1. In the simulation, we randomly

chose NCellBT cells to form the successfully sequenced pre-treatment sample whose lineage label information was further used to classify pre-sensitive and pre-resistant cells.

For the other half of the sample that subsequently underwent anti-cancer treatment, the “true pre-sensitive” cells were removed while “true pre-resistant” cells were selected to form the surviving population in the simulation. After treatment, the surviving cells were cultured for recovery stage. The number of cells after the recovery stage was defined as a parameter NCellPreSeq, ranging from 51,000 to 290,000 for the three treatment conditions. In the simulation, we duplicated surviving cells to reach the number of NCellPreSeq. Those cells were sampled and sequenced to extract lineage label information. Due to scRNA-seq’s limited loading capacity, not all the recovered cells were loaded into the sequencing device. We defined a parameter called NCellSeq to be the number of cells loaded for scRNA-seq, which was 10,000 for all treatment conditions. In the simulation, we randomly chose NCellSeq cells from those NCellPreSeq cells. During the scRNAseq, quality control resulted in further drop-out of the cells. In the simulation we randomly selected NCellAT cells to form the successfully sequenced post-treatment sample. For the parameters NCellPreSeq, NCellSeq, and NCellAT, we estimated their values directly from the scRNA-seq data from the actual experiment as shown in Supplementary Data 1.

In the end, we obtained two samples with lineage label information from the scRNA-seq data: the NCellBT cells in the pre-treatment sample and the NCellAT cells in the post-treatment sample. One of the objectives for ReSisTrace experiment is to classify the cells of the pre-treatment sample into pre-resistant and pre-sensitive groups. To achieve that, we relied on the lineage label information from the post-treatment sample, as all of them were considered as true resistant cells since they survived the drug treatment. Namely, in the pre-treatment sample, the cells with lineage label which was found in the post-treatment sample were predicted as pre-resistant while the others were predicted as pre-sensitive cells.

The pre-resistant predictive rate, defined as the proportion of true pre-resistant cells in the predicted pre-resistant cells, should always be 100%, because all the lineages found in the post-treatment sample are true resistant cells according to our definition. In contrast, there exists uncertainty when predicting the pre-sensitive cells, due to several random events such as i) the cells not doubling in the doubling stage (accommodated into the LineagePropAfterDoubling parameter in simulation), ii) the sisters not being evenly distributed in the splitting stage (taken into account by random sampling of cells in the simulation), or iii) cell drop outs during sample processing, sequencing and quality control (accommodated into the NCellPreSeq, NCellSeq, and NCellAT parameters in the simulation). In particular, we were interested in the relationship of the pre-sensitive predictive rate, defined as the proportion of true pre-sensitive cells in the predicted pre-sensitive cells, as a function of killing rate of treatments (i.e. KillRateTotal). The results from the simulations under experimental conditions are shown in Supplementary Fig. 1h (Carboplatin in blue line; Olaparib in red line and NK in brown line). To estimate the upper bound of the pre-sensitive predictive, we performed an additional simulation under the perfect condition where i) all the cells are perfectly doubled in the doubling stage (LineagePropAfterDoubling: doubled = 100%, quadrupled = 0%, singleton = 0%), and ii) no drop out during sample processing and sequencing (NCellBT = NCellAT = NCellPreSeq = NCellSeq = SampleSize = 16,000). The result is shown in Supplementary Fig. 1h in black line.

Supplementary Methods

Cell cycle flow cytometry analysis

Cells were fixed by slowly adding ice-cold 70% ethanol and keep at at 4°C (or -20°C) for >4h. Cells were spin down (500g, 5 min) and the pellet washed once with PBS + 2% FBS, then treated with RNase A (100 µl RNase A for one million cells at a concentration of 1 mg/ml) and incubated at 37°C for 30 min. Cells were then stained with 25 µg/ml PI (Thermo Fisher, P3566) for ≥30 min at RT protected from light. Sample quantification was performed with flow cytometry NovoCyte Quanteon Analyser (three replicates per condition). Gating strategies shown in Supplementary Fig. 12.

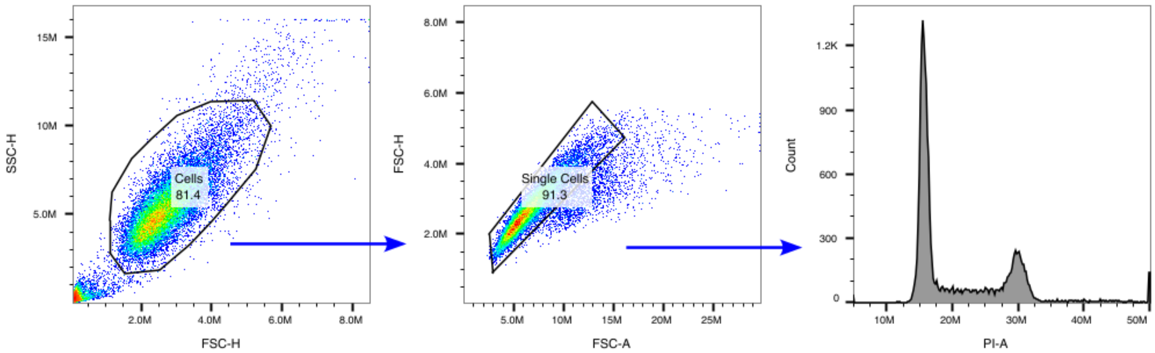

Supplementary Fig. 12 Gating strategy used for the flow cytometry analysis.
