# Supplementary material for: Tracing back primed resistance in cancer via sister cells
Source: Nat Commun. 2024 Feb 7;15:1158. doi: 10.1038/s41467-024-45478-7 (PMC10850087; doi:10.1038/s41467-024-45478-7)
Supplement: Supplementary file 3 — Description of Additional Supplementary Files [file 41467_2024_45478_MOESM3_ESM.pdf]

Supplementary Data 1. Cell counts from scRNA-seq experiments for determining the parameters in the computer simulations.

Supplementary Data 2. Pre-resistance gene expression signatures for the treatments, control condition and the CNV subclones.

Supplementary Data 3. Full list of significant GO annotations for pre-resistance and subclonal signatures.

Supplementary Data 4. HRD signatures from COV362 and TCGA clinical cohort.

Supplementary Data 5. HLA-C haplotypes of the utilised NK cells and ovarian cancer cell lines.

Supplementary Data 6. NK transcriptional signature.

Supplementary Data 7. Predicted drugs for sensitising the intended treatments.

Supplementary Data 8. Expression of the drug targets in Kuramochi cells.

Supplementary Data 9. DNA sequence of pBA439_UMI20 plasmid.

Supplementary Data 10. Primers for experimental BRCAness model constructs.

Supplementary Data 11. Cell number of all samples from the ReSisTrace experiments.

Supplementary Data 12. Code for implementing the simulations.

Supplementary Data 13. Concentrations of small molecules used in drug-induced transcriptome profiles by scRNA-seq experiment.

Supplementary Data 14. Thresholds for the scRNA-seq data quality control.
