# Supplementary material for: Tracing back primed resistance in cancer via sister cells
Source: Nat Commun. 2024 Feb 7;15:1158. doi: 10.1038/s41467-024-45478-7 (PMC10850087; doi:10.1038/s41467-024-45478-7)
Supplement: Supplementary file 18 — Reporting Summary [file 41467_2024_45478_MOESM18_ESM.pdf]

nature portfolio

Corresponding author(s): Anna Väharautio
Jing Tang

Last updated by author(s): Dec 22, 2023

Reporting Summary

Nature Portfolio wishes to improve the reproducibility of the work that we publish. This form provides structure for consistency and transparency in reporting. For further information on Nature Portfolio policies, see our Editorial Policies and the Editorial Policy Checklist.

Statistics

For all statistical analyses, confirm that the following items are present in the figure legend, table legend, main text, or Methods section.

n/a	Confirmed	
☐	☒	The exact sample size (*n*) for each experimental group/condition, given as a discrete number and unit of measurement
☐	☒	A statement on whether measurements were taken from distinct samples or whether the same sample was measured repeatedly
☐	☒	The statistical test(s) used AND whether they are one- or two-sided<br>*Only common tests should be described solely by name; describe more complex techniques in the Methods section.*
☐	☒	A description of all covariates tested
☐	☒	A description of any assumptions or corrections, such as tests of normality and adjustment for multiple comparisons
☐	☒	A full description of the statistical parameters including central tendency (e.g. means) or other basic estimates (e.g. regression coefficient) AND variation (e.g. standard deviation) or associated estimates of uncertainty (e.g. confidence intervals)
☐	☒	For null hypothesis testing, the test statistic (e.g. *F*, *t*, *r*) with confidence intervals, effect sizes, degrees of freedom and *P* value noted<br>*Give P values as exact values whenever suitable.*
☒	☐	For Bayesian analysis, information on the choice of priors and Markov chain Monte Carlo settings
☒	☐	For hierarchical and complex designs, identification of the appropriate level for tests and full reporting of outcomes
☐	☒	Estimates of effect sizes (e.g. Cohen's *d*, Pearson's *r*), indicating how they were calculated

Our web collection on statistics for biologists contains articles on many of the points above.

Software and code

Policy information about availability of computer code

Data collection	Single cell RNA sequencing was performed by using the 10X Genomics Chromium Next GEM Single Cell 3' kit v3.1 according to the manufacturer's instructions. Libraries were sequenced on Illumina NovaSeq 6000.
Data analysis	The software used in for data analysis in this study: CellRanger (v5.0.1), UMI-tools (v1.0.1), velocyto (v0.17.17), scVelo (v0.2.3), Seurat R package (v4.0.4), vegan R package (v2.6.2), distance R package (v 0.1.8), InferCNV R package (v1.7.1), limma R package (v3.46.0), survival R package (v3.3.1), clusterProfiler (v3.18.1), PRISM (v0.9), Enrichr (update on March 29th, 2021), synergyfinder R package (v3.4.5), PharmacoGx R package (v3.0.2), UCell R package (1.3.1). The source code for data analysis will be available at GitHub (https://github.com/TangSoftwareLab/ReSisTrace) and Zenodo (https://doi.org/10.5281/zenodo.10418352).

For manuscripts utilizing custom algorithms or software that are central to the research but not yet described in published literature, software must be made available to editors and reviewers. We strongly encourage code deposition in a community repository (e.g. GitHub). See the Nature Portfolio guidelines for submitting code & software for further information.

Data

Policy information about availability of data

All manuscripts must include a data availability statement. This statement should provide the following information, where applicable:

- Accession codes, unique identifiers, or web links for publicly available datasets
- A description of any restrictions on data availability
- For clinical datasets or third party data, please ensure that the statement adheres to our policy

All the data needed to evaluate the conclusions in the paper are available in the manuscript or the supplementary materials. All of the ReSisTrace scRNA-seq raw data have been deposited in the Gene Expression Omnibus (GEO) under accession code GSE223003. Source data are provided with this paper.

Research involving human participants, their data, or biological material

Policy information about studies with human participants or human data. See also policy information about sex, gender (identity/presentation), and sexual orientation and race, ethnicity and racism.

Reporting on sex and gender	N/A
Reporting on race, ethnicity, or other socially relevant groupings	N/A
Population characteristics	N/A
Recruitment	N/A
Ethics oversight	N/A

Note that full information on the approval of the study protocol must also be provided in the manuscript.

Field-specific reporting

Please select the one below that is the best fit for your research. If you are not sure, read the appropriate sections before making your selection.

☒ Life sciences ☐ Behavioural & social sciences ☐ Ecological, evolutionary & environmental sciences

For a reference copy of the document with all sections, see nature.com/documents/nr-reporting-summary-flat.pdf

Life sciences study design

All studies must disclose on these points even when the disclosure is negative.

Sample size	ReSisTrace was performed on two samples per treatment or control. Drug-perturbed scRNA-seq profiles were similarly obtained from two replicates for each treatment or control, and other assays with three replicates. For ReSisTrace the comparisons are first performed on cell populations within individual sample, reducing any batch effects. The two replicates were highly concordant and thus enable robust analysis.
Data exclusions	No samples or replicates were excluded.
Replication	Two replicates were performed for each ReSisTrace experiment and drug-perturbed scRNA-seq profile per treatment or control. At least three replicates were used for other in vitro experiments. All replicates are successful.
Randomization	Lineage labeled cells were divided from a common pool to random aliquots for the experiments.
Blinding	Analysis was performed on unbiased measurement approaches (scRNA-seq, luminescence for cell viability), and hence blinding was not relevant.

Reporting for specific materials, systems and methods

We require information from authors about some types of materials, experimental systems and methods used in many studies. Here, indicate whether each material, system or method listed is relevant to your study. If you are not sure if a list item applies to your research, read the appropriate section before selecting a response.

Materials & experimental systems

n/a	Involved in the study
☐	☒ Antibodies
☐	☒ Eukaryotic cell lines
☒	☐ Palaeontology and archaeology
☒	☐ Animals and other organisms
☒	☐ Clinical data
☒	☐ Dual use research of concern
☒	☐ Plants

Methods

n/a	Involved in the study
☒	☐ ChIP-seq
☐	☒ Flow cytometry
☒	☐ MRI-based neuroimaging

Antibodies

Antibodies used

Primary antibodies:
RAD51 (ab133534, Abcam, diluted 1:1000)
cyclin A2 (GTX634420, GeneTex, diluted 1:500)

Secondary antibodies:
goat anti-mouse IgG-Alexa Fluor 488 (A11029, LifeTechnologies, diluted 1:1000)
goat anti-mouse IgG-Alexa Fluor 568 (A11004, LifeTechnologies, diluted 1:1000)
goat anti-rabbit IgG-Alexa Fluor 647 (A21245, LifeTechnologies, diluted 1:1000)

Validation

Commercial antibodies are validated by the supplier.

Eukaryotic cell lines

Policy information about cell lines and Sex and Gender in Research

Cell line source(s)

293FT cells (Thermo Fisher, R70007); COV362 cells (Merck, 07071910); KURAMOCHI (JCRB Cell Bank, JCRB0098); UWB1.289 (ATCC, CRL-2945); UWB1.289+BRCA1 (ATCC, CRL-2946).

Authentication

Cell line authentication was performed with the Promega GenePrint 10 system, which allows co-amplification and detection of ten human loci (9 STR loci and Amelogenin for gender identification) at Genotyping Unit of Technology Centre, Institute for Molecular Medicine Finland (FIMM).

Mycoplasma contamination

All cell lines used were mycoplasma negative as tested by LookOut® Mycoplasma qPCR Detection Kit (Merck, MP0035-1KT).

Commonly misidentified lines
(See ICLAC register)

No commonly misidentified cell lines were used.

Plants

Seed stocks

N/A

Novel plant genotypes

N/A

Authentication

N/A

Flow Cytometry

Plots

Confirm that:

☒ The axis labels state the marker and fluorochrome used (e.g. CD4-FITC).

☒ The axis scales are clearly visible. Include numbers along axes only for bottom left plot of group (a 'group' is an analysis of identical markers).

☒ All plots are contour plots with outliers or pseudocolor plots.

☒ A numerical value for number of cells or percentage (with statistics) is provided.

Methodology

Sample preparation	Cells were fixed by slowly adding ice-cold 70% ethanol and keep at at 4°C (or -20°C) for >4h. Cells were spin down (500g, 5 min) and the pellet washed once with PBS + 2% FBS, then treated with RNase A (100 µl RNase A for one million cells at a concentration of 1 mg/ml) and incubated at 37°C for 30 min. Cells were then stained with 25 µg/ml PI (Thermo Fisher, P3566) for ≥30 min at RT protected from light.
Instrument	NovoCyte Quanteon Analyser
Software	NovoExpress flow cytometry software
Cell population abundance	Cell sorting not employed
Gating strategy	Using the FSC/SSC gating, debris and cell clusters were removed by gating on the main cell population.

☒ Tick this box to confirm that a figure exemplifying the gating strategy is provided in the Supplementary Information.
